# AI-Powered Framework for Personalized Prescription of Physical Activity in Aging: Proposing PEPHA, a framework for Personalized Phenotyping for Aging

**DOI:** 10.2196/95123

**Published:** 2026-10-06

**Authors:** Igor Matias, Melanie Mack, Matthias Kliegel, Katarzyna Wac

**Affiliations:** 1 Quality of Life Technologies Lab, University of Geneva, Route de Drize 7, Battelle A, Carouge, Geneva, 1227, Switzerland, 41 223790234; 2 Cognitive Aging Lab, University of Geneva, Geneva, Switzerland; 3 Center for the Interdisciplinary Study of Gerontology and Vulnerabiity, University of Geneva, Geneva, Switzerland

**Keywords:** artificial intelligence, digital phenotyping, wearables, physical activity, cognitive aging, processing speed, depression, personalized intervention, temporal modeling, explainable AI

## Abstract

**Background:**

Personalizing physical activity recommendations for older adults requires understanding not only which dimensions of physical activity and sedentary behaviors (24-h movement behaviors) influence health outcomes but also when, within an individual’s everyday life, these dimensions are most relevant. Current observational and interventional approaches rarely capture the temporal dynamics linking everyday patterns of 24-hour movement behaviors to cognitive and mental health trajectories, 2 key determinants of healthy aging.

**Objective:**

This study introduces and evaluates PEPHA (Personalized Phenotyping for Aging), an interpretable artificial intelligence (AI) framework designed to identify which dimensions of behavior and when within an observation window are strongly associated with cognitive functioning and depressive symptoms in older adults.

**Methods:**

We introduce PEPHA, an interpretable AI framework that integrates passive, high-frequency wearable data (physical activity and sleep) with periodic, active, validated cognitive and affective assessments (waves). Using longitudinal data from the *Providemus alz* cohort (n=67, up to 6 waves and 528 days of wearable data per person), we examined 2 key outcomes representing cognitive functioning and mental health (processing speed and depressive symptoms). PEPHA summarizes daily physical activity and sedentary patterns into slope-based temporal features, optimizes support vector regression parameters through Bayesian optimization, and applies individualized analyses including time order-swap testing and change point detection to identify “potential temporal association windows” (ie, periods within an observation window during which outcomes appear more sensitive to changes in behavioral patterns).

**Results:**

Personalized analyses showed that roughly 40% of individuals exhibited moderate or large temporal order effects of 24-hour movement behavior in the outcomes. For 1 exemplar participant (male, above the mean sample age), we localized 2 potential temporal association windows approximately 60 days and 30 days before the assessment of his processing speed, suggesting periods during which this individual may have been more sensitive to favorable or unfavorable behavioral configurations. Across participants, PEPHA revealed distinct 24-hour movement behavioral patterns correlated with cognitive functioning and mental health. Processing speed was best explained by locomotor activity, while depressive symptoms were best explained by sedentary behavior. Five control variables (education, cognitive reserve, diet, sex, and subjective age difference) were noninformative, whereas chronological age had predictive power regarding depressive symptoms.

**Conclusions:**

PEPHA demonstrates that continuous passive wearable data can uncover individualized, time-specific behavioral patterns associated with cognitive functioning and mental health. Although exploratory, this framework transforms observational data into interpretable, timing-aware insights that can help identify periods of increased behavioral sensitivity or association, thereby informing future personalized, AI-supported physical activity interventions in aging.

## Introduction

Physical activity (PA) is widely recognized as one of the most effective strategies to promote healthy aging, with robust evidence from cross-sectional, longitudinal, and intervention studies demonstrating benefits across multiple domains, including cognitive functioning and mental health [1-3]. Guidelines currently emphasize standardized thresholds (eg, minutes of moderate activity per week [4]). However, this “one-size-fits-all” approach does not encompass the high inter- and intra-individual differences in the aging process. Individuals differ not only in baseline characteristics and responsiveness to PA (interindividual differences), but they also show dynamic fluctuations in outcomes over time (intra-individual differences) [5,6]. Additionally, they may also differ in how the timing (when) and dynamics (how) of behavioral change influence their outcomes. For example, the association between moderate-to-vigorous PA and mental health outcomes may vary depending on the delay (potential temporal association window) between changes in activity and outcome assessment, reflecting unknown latency (when) and impact (how) of behavioral effects over time. Clinically, this should not be interpreted as prescribing a behavior at an exact number of days before an assessment but rather as identifying periods during which outcomes may be more sensitive or vulnerable to favorable or unfavorable behavioral patterns. Nevertheless, understanding when and how the dynamics of an individual’s PA and sedentary behaviors relate to key outcomes of healthy aging is central to advancing precision approaches in PA prescription for older adults.

Observational approaches are particularly important in this field because they capture real-world behavior in naturalistic contexts, outside the constraints of structured interventions [7]. Unlike randomized trials, which typically prescribe a fixed regimen, observational cohorts enable researchers to examine natural variations in 24-hour movement behavior as well as their associations with changes in cognitive functioning and mental health [8]. Such data are essential for developing methods that can later inform and personalize interventions.

Recent advances in wearable technology now enable continuous, passive, multidimensional monitoring of 24-hour movement behavior, such as sleep, PA, and sedentary time, across weeks, months, and even years [9] in everyday environments like academic ones [10,11]. In a similar way, advances in mobile computing allow for ubiquitous and repeated active episodic assessments (in waves) of outcomes of central importance for aging populations, such as cognitive functioning and mental health [12,13]. These developments create an opportunity for data-driven personalization of PA recommendations but require frameworks that can relate dense, high-frequency behavioral data to relatively sparse outcome assessments while capturing both distributional patterns and temporal dynamics.

The concept of digital phenotyping, defined as using continuous sensor data to characterize behavior and health states, has become central to precision health research [14,15]. Large-scale cohorts such as ActiveAgeing [16] and All of Us [17] integrate wearable sensing with clinical assessments to study autonomy and functional decline, yet most analyses focus on risk stratification (eg, fall risk, frailty) or cross-sectional prediction at the population level rather than inter- and intra-individual longitudinal dynamics. Woll et al [18] systematically reviewed machine learning approaches for mental health monitoring via wearable and sensor data and concluded that current models lack adequate personalization and poorly capture intra-individual variability. Despite some progress in artificial intelligence (AI)–powered personalization of PA programs [19,20], substantial limitations remain in health condition specificity, adaptivity, and long-term personalization [19,21-23], particularly on an intra-individual level in determining when outcomes may be most sensitive to changes in 24-hour movement behavior [24]. This underscores the need for AI frameworks capable of translating continuous passive data into individualized, time-aware behavioral insights.

This has motivated new individualized modeling methods sensitive to behavioral timing. The model-twin randomization (MoTR) framework [25] exemplifies this trend by enabling intra-individual causal inference with AI models as personalized simulators to estimate each participant’s average treatment effect: that is, the expected outcome difference between their observed behavior and a randomized behavioral alternative. However, although MoTR requires no experimental interventions, it still relies on dense, high-frequency measurement of both predictors and outcomes, an assumption seldom feasible in cognitive functioning and mental health research, where validated cognitive tasks and mental questionnaires are effortful and typically spaced weeks or months apart.

Additionally, a major challenge in AI-driven health research is balancing predictive accuracy with interpretability. As Doshi-Velez and Kim [26] argued, interpretable models are essential for trustworthy clinical translation. Many high-performing systems reveal associations between 24-hour movement behaviors and outcomes but fail to indicate when behavioral sequences matter or how timing influences effectiveness. Moreover, causal inference in observational data remains inherently limited. As Pearl and Mackenzie [27] emphasized, correlation alone cannot establish causal direction without explicit assumptions.

To address these gaps, we introduce PEPHA (Personalized Phenotyping for Aging), an interpretable AI framework designed to identify both which 24-hour movement behavioral dimensions and when their changes are potentially most relevant to cognitive functioning and mental health. Unlike traditional predictive models that focus on identifying individuals at risk of cognitive or mental health decline, PEPHA is designed to reveal how patterns of behavior potentially relate to cognitive functioning and mental health within a given individual over time. Rather than inferring causality, it summarizes behavioral dynamics into interpretable, timing-sensitive patterns that can support personalization of possible future interventions.

Building on data from the *Providemus alz* study [28], an ongoing longitudinal cohort of community-dwelling older adults monitored with consumer-grade wearables and quarterly cognitive-mental assessments, we demonstrated PEPHA’s proof-of-concept application. Specifically, we answered 2 research questions (RQs): (RQ1) To what extent do different dimensions of 24-hour movement behavior predict cognitive and mental outcomes in adults? and (RQ2) How does the timing of behavioral change influence these associations for a specific individual, and can individualized “potential temporal association windows” be identified within each observation period? The primary objective of PEPHA is not to re-establish associations between behavior and the selected outcomes but to provide an interpretable framework capable of identifying when behavioral patterns appear potentially most relevant for a given individual.

To answer these questions, PEPHA combines 3 analytic layers: (1) slope-based summarization of behavioral trajectories over short time windows, (2) lag-based order-swap testing to quantify whether associations differ as a function of the temporal lag between behavioral change and outcome assessment, and (3) change point detection to localize the specific timing of behavioral changes potentially associated with variability in cognitive functioning and mental health outcomes. This unified framework addresses the mismatch between continuous behavioral monitoring and intermittent outcome measurement by providing a structured pathway from population-level modeling to individual-level identification of temporal potential temporal association windows. As a proof of concept, we focused on 2 clinically relevant aging outcomes—processing speed and depressive symptoms—which represent cognitive and mental dimensions of healthy aging. Applying PEPHA to these outcomes, we illustrated how continuous real-world data can be repurposed to inform personalized, timing-aware understanding of PA patterns. Ultimately, this framework advances the broader goal of AI-supported approaches to PA in aging by shifting the focus from what activity is performed toward when specific behavioral adjustments may be most relevant.

## Methods

### Ethical Considerations

All procedures were approved by the Commission Cantonale d’Ethique de la Recherche sur l’être humain with the number 2023‐00975. Written informed consent was obtained from all participants before starting data collection. Participants received no monetary compensation.

### Data Sources and Population

Data used in this work came from the Providemus alz project [28], an ongoing observational cohort of community-dwelling adults (45.61‐77.62 years old), being conducted at the University of Geneva, Switzerland. Participants (cognitively healthy individuals) volunteered to wear a consumer-grade smartwatch (Withings Steel HR) continuously and actively complete wave-based assessments approximately quarterly (every 90 days) over 2 years using a custom-made mobile app (mQoL, based on the mQoL Living Lab infrastructure [29]) available for iOS and Android devices. Data collection started in March 2024 and lasted until April 2026.

The provided smartwatch was used to collect technology-reported outcomes (like PA, heart rate, and sleep) passively (referred to as passive data in the rest of this paper). Patient-reported outcomes (like depressive symptoms) and performance-reported outcomes (like processing speed) were actively obtained (referred to as active data in the rest of this paper) via an interaction with the participant through the mQoL app available for iOS and Android devices.

For this paper, we used Providemus alz data collected until August 20, 2025, corresponding to a maximum of 6 repeated assessments using validated questionnaires and tests (waves) and a maximum of 528 days of wearable data per participant. Figure 1 illustrates the data collection timeline.

**Figure 1.**
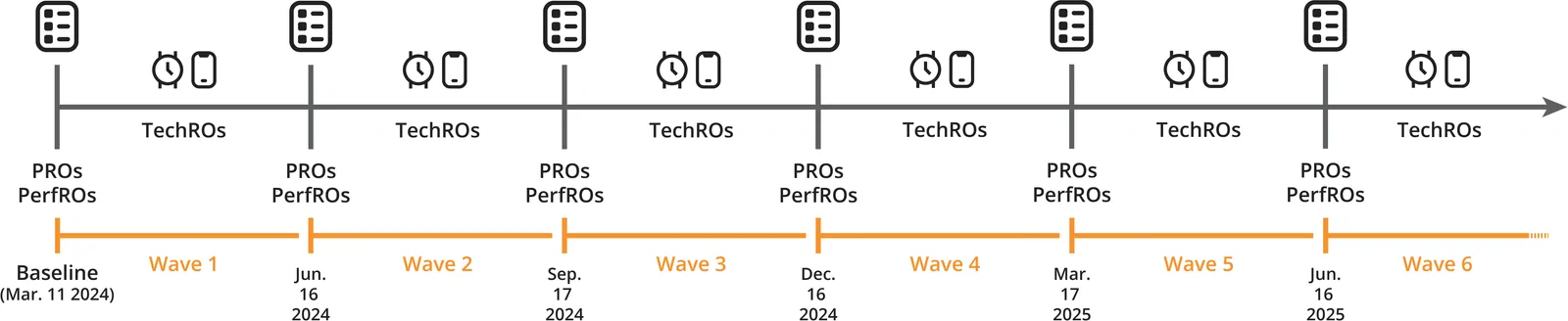
Timeline of the collection of data used in this research. PerfRO: performance-reported outcome; PRO: patient-reported outcome; TechRO: technology-reported outcome.

Following the data quality criteria applied in previous research [30,31], data from 67 participants were included in the analysis (Table 1 provides the baseline characteristics). Validated assessments covered 20 cognitive functioning and mental health outcomes, but in this paper, we focused only on 2 clinically relevant measures: processing speed (measured using the Trail Making Test [32] part A) and depressive symptoms (a suboutcome of the Hospital Anxiety and Depression Scale [HADS] [33]).

**Table 1.** Baseline characteristics of the population studied.

Characteristics	Results (n=67)
Sex at birth, n (%)	
Male	25 (37)
Female	42 (63)
Age at enrollment (years), mean (SD)	58.40 (8.79)
Age at enrollment (years), range	45.61‐77.62
Ethnic origin	
White	64 (96)
Asian	2 (3)
Other	1 (1)
Native language used during the study	
Yes	60 (90)
No	7 (10)
Education (years, reported across waves), mean (SD)	17.32 (4.36)
Education (years, reported across waves), range	6.00‐31.60
Total CRIq^a^ [34] score, mean (SD)	127.81 (19.35)^b^
Total CRIq score, range	92.00‐197.00
MDS^c^^,^^d^ [35], mean (SD)	32.58 (4.10)
MDS, range	24.00‐41.00

a CRIq: Cognitive Reserve Index questionnaire.

b Medium-high.

c MDS: Mediterranean diet score.

d Scale range: 0-55.

### Measures

In total, 29 variables served as predictors and were sampled at 2 frequencies (daily and once per wave), as presented in Textbox 1.

Textbox 1. **Listing of predictors used.**

Sampled daily (22 variables)

- Locomotor activity (2 variables):
  - Steps per second wearing the watch (step frequency)
  - Meters per second wearing the watch (distance frequency)
- 24-hour heart rate (HR) dynamics (8 variables):
  - Mean (bpm)
  - SD (bpm)
  - Median (bpm)
  - IQR (bpm)
  - Minimum (bpm)
  - Maximum (bpm)
  - Skewness
  - Kurtosis
- Intensity-specific physical activity (PA) duration (7 variables):
  - Seconds spent in soft activity [36]
  - Seconds spent in moderate activity
  - Second spent in intense activity
  - Seconds spent in HR zone 1
  - Seconds spent in HR zone 2
  - Seconds spent in HR zone 3
  - Minutes spent in moderate and vigorous activity (MVPA)
- Sedentary behavior (2 variables):
  - Seconds spent in HR zone 0
  - Minutes wearing the watch and not engaging in soft, moderate, or intense activity
- Controls (3 variables):
  - Percentage of the day wearing the watch
  - Percentage of time windows missing in the fit
  - Sleep regularity index (0 to 100)

Sampled once per wave (7 variables)

- Controls (7 variables)
  - Total Cognitive Reserve Index questionnaire (CRIq) score
  - Mediterranean diet score (MDS) score
  - Chronological age (years)
  - Difference between subjective and chronological ages (years)
  - Sex at birth
  - Education years
  - Number of assessment tries

For the predictors sampled daily, wear time was defined in a previous publication [28]. The percentage of missing windows per day was used so the model could account for the incompleteness of some linear or quadratic fits (introduced later in this section). The sleep regularity index quantified night-to-night consistency in sleep-wake timing; it captures temporal regularity of sleep behavior, computed by comparing sleep-wake states across consecutive days in 15-minute time bins and adjusting for time zone shifts.

For the predictors sampled once per wave, Cognitive Reserve Index questionnaire (CRIq) and Mediterranean diet score (MDS) values were only obtained at baseline and, therefore, had a constant value across all waves. Chronological age was computed based on the date of the active assessment. The difference between subjective and chronological age was obtained by first asking the participants at every wave to select the decade with which they identified the most then subtracting its midpoint value from the chronological age (negative values indicating feeling older than their actual age). The “number of assessment tries” was used to account for the cases in which, for technical or personal reasons, participants started to complete the assessments but did not finish them, having another try some time later in the same wave; it served as an indirect measurement of potential learning effect within a wave. At the end, 29 variables were used as predictors.

The 2 outcomes studied, processing speed and depressive symptoms, were collected once per wave and matched with daily and once-per-wave data while preserving temporal order. Baseline characteristics of both outcomes across all the data used in this research are provided in Table 2. For each outcome measured on day *D*, the analysis included only predictor data (daily and once-sampled) recorded before *D*.

No data imputation was performed on either the predictors or the outcomes at any stage of the analysis; all models were trained and evaluated using only the available observed data.

**Table 2.** Baseline characteristics of the outcomes studied.

Characteristics	Processing speed	Depressive symptoms (abnormal case if>10)
Mean (SD)	3.964 (2.957)	5.264 (2.330)
Median (IQR)	3 (4)	5 (2)
Linear trends, mean (SD)	–0.171 (0.620)	–0.175 (1.043)
Quadratic trends, mean (SD)	0.039 (0.816)	0.009 (0.761)
Quadratic trends value, %		
Positive	45.45	52.24
Zero	3.03	2.99
Negative	51.52	44.78

### PEPHA Framework Conceptualization

To address the study objectives concerning the relationships among 24-hour movement behaviors, the temporal dynamics, and cognitive functioning and mental health outcomes, we applied the PEPHA framework. It is an adaptable analytic approach designed for observational and ecological settings, enabling the integration of high-frequency wearable data with lower-frequency, validated outcome measures. By leveraging naturally occurring behavioral variation, the framework characterizes, at both population and individual levels, which behavioral dimensions are potentially associated with a given outcome and how these associations vary across time within an observation period based on historical individual data.

The framework was implemented using a regressive support vector machine (SVM) with a radial basis function (RBF) kernel. This model was selected for its robustness in moderate sample sizes, ability to capture nonlinear associations, and stability in high-dimensional feature spaces (characteristics typical of longitudinal aging datasets derived from wearable sensors). Within PEPHA, the SVM is not used for outcome optimization or causal inference but as a stable function approximator that enables systematic comparison of behavioral representations across time windows, lag structures, and individuals. All framework parameters are generic and configurable (eg, wave duration), allowing replication and adaptation to other datasets and outcomes through the accompanying open-source implementation [37].

### Data Alignment and Inclusion

PEPHA begins by aligning high-frequency passive data with lower-frequency outcome assessments. Each outcome collected at wave W+1 is paired with the passive data recorded during the 90 days preceding it (wave W), so that predictors always refer to the 24-hour movement behaviors before the outcome. As the assessment day of each outcome can differ, that 90-day window is dynamically computed depending on the outcome being studied at a time. An illustration of this alignment is in Figure 2.

**Figure 2.**
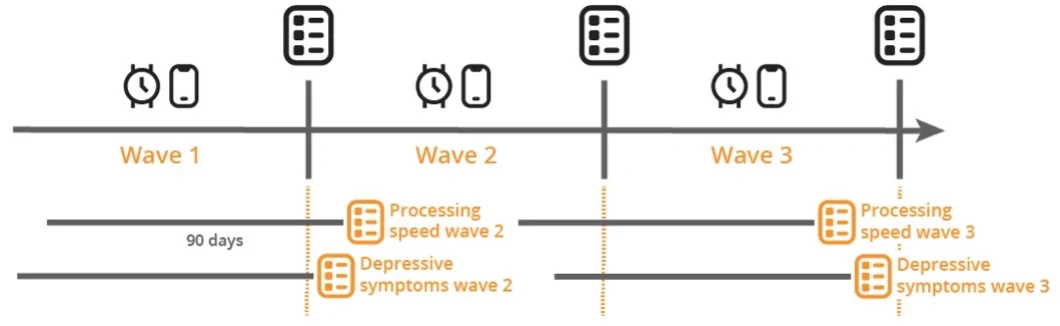
Diagram illustrating the alignment of passive data with outcome assessments, given a flexible wave for each participant.

The framework uses two key parameters at this stage: (1) *TARGET_DAYS*, which defines the retrospective window length (eg, 90 d), and (2) *MIN_VALID_WAVES,* which defines the minimum number of valid waves required for a participant to be included in the next steps.

A wave for a specific participant is considered valid if (1) at least 50% of its daily data are available (defined as having at least 10 h of wear time, as in [31]) and (2) the outcome value at W+1 is present.

In the Providemus alz application, these rules ensured that only consistent and interpretable wave-outcome pairs were retained, yielding a robust set of participant-wave samples for modeling. Given the outcome assessment under noncontrolled mobile settings, processing speed results were only considered valid if within the mean ±2.5 SD of the data initially collected. Depressive symptoms scores were not filtered, as they were assessed regardless of completion times and using a standardized and validated questionnaire.

Following our previous analyses of the Providemus alz cohort [31], we tested the modeling of processing speed and depressive symptoms using *levels* (absolute scores) and *absolute drifts* (deviations from prior medians), given the differences found earlier. This distinction reflects the 2 temporal perspectives that PEPHA can capture. In the *levels* variant, the model uses the temporal slopes of daily predictors during the 90 days preceding each assessment to predict the state of the outcome at that assessment (eg, how recent [90 d] fluctuations in PA or sedentary lifestyle relate to concurrent cognitive or affective levels). In the *drift* variant, the model instead links the change in outcome values between consecutive waves to the patterns of change in 24-hour movement behavioral dynamics within each 90-day window, thus focusing on how habits changes accompany longer-term shifts in brain-health trajectories.

For the methods of this research and following the data inclusion criteria described earlier, the *levels* models were trained on data from 66 participants (average per participant: 4.67, SD 0.69) for processing speed and from 67 participants (average per participant: 4.52, SD 0.82) for depressive symptoms. The *drift* models, in contrast, included 55 participants (average per participant: 4.91, SD 0.29) for processing speed and 54 participants (average per participant: 4.76, SD 0.58) for depressive symptoms. This difference reflects the fact that *drift* models require 2 consecutive waves to estimate within-person change and therefore begin at wave 3, whereas *levels* models begin at wave 2, which meant participants who stopped adhering to the study’s protocol before wave 3 could not be modeled in the *drift* case.

### Windowing and Trend Features From High-Frequency Daily Data

Once valid daily data are selected, PEPHA transforms them into compact temporal summaries to reduce the influence of short-term extremes and noise. The 90-day period is divided into consecutive, nonoverlapping windows of *WINDOW_DAYS* (typically between 2 and 7), balancing the need for sufficient observations to compute reliable summary statistics with the ability to capture short-term behavioral variation. Each window is summarized as a 24-hour movement behavioral “snapshot” from which 8 descriptive statistics are then computed: mean, median, SD, IQR, minimum, maximum, skewness, and kurtosis. This produces a temporal sequence of windows for every predictor.

The sequence of descriptive statistics is then split into 2 halves—early (Half1) and late (Half2)—corresponding to the first and second halves of the observation period between the waves (45 d each). For each half, PEPHA fits both linear and quadratic regressions to estimate the trend slopes that summarize change over time. Optionally, the SE of each slope can also be computed to account for trend fit, controlled by a parameter in the framework referred to as *USE_SE*. The result is a structured set of slope-based features describing not only how much each 24-hour movement behavioral variable changes (linear) but also how within the wave these changes occur (quadratic).

In PEPHA and across both *levels* and *absolute drift* variants, the model learns from temporal trends (relying on predictors being the linear and quadratic fits) rather than static levels (eg, having access to the actual sequence of PA data), emphasizing how 24-hour movement behaviors evolve rather than how high or low they are. This design allows PEPHA to identify which behavioral dimensions and which temporal patterns best explain individual differences in outcomes, independent of baseline offsets.

For the example application of the framework here presented, no data imputation was applied; missing values were retained throughout the pipeline, preserving the natural irregularities of real-world behavior. Regressions were fitted directly on the available data, respecting the temporal spacing of missing windows rather than interpolating them. The resulting features provide a compact and interpretable representation of temporal behavior for each wave.

### Optuna-Based Parameter Optimization

Because PEPHA integrates multiple analytical components (temporal windowing, regression feature extraction, and support vector modeling), the performance of its modeling phase depends on several interacting complexity parameters. Selecting these parameters manually or through grid search would be inefficient and prone to suboptimal results. To ensure reproducibility and avoid arbitrary choices, PEPHA uses Bayesian optimization via Optuna [38], an adaptive search algorithm that iteratively explores the parameter space to identify hyperparameter combinations that balance predictive accuracy, correlation strength, and variance fidelity under a consistent evaluation procedure.

Parameters jointly optimized include the following. *MIN_VALID_WAVES* is the minimum number of valid waves per participant to be included in the modeling and analysis. *WINDOW_DAYS* is the length of each summarization window in the predictors data. *USE_SE* indicates whether SEs of the fitted linear and quadratic trends are used as predictors aside from the trends themselves. *CORR_THRESHOLD* is the maximum absolute correlation allowed between redundant predictors before one is dropped; it is applied to avoid information repetition, as detailed later in this section. *SVR_C*, *SVR_epsilon*, and *SVR_gamma* are core regressive SVM model hyperparameters controlling regularization, insensitivity margin, and kernel width.

Each Optuna trial performs 10-fold grouped cross-validation (grouped by participant to make sure one participant can never have data in both train and test sets at the same time, which would represent a data leakage case) and returns a single objective score that integrates 3 complementary criteria:

$Objective=0.5\text{\textbackslash{} }\times \text{\textbackslash{} }MAEz\text{-}1.2\text{\textbackslash{} }\times \text{\textbackslash{} }r\text{+}0.5\text{\textbackslash{} }\times \text{\textbackslash{} }|\frac{\sigma pred}{\sigma true}\text{-}1|$(1)

where *MAEz* is the mean absolute error (MAE) normalized by the SD of the true outcomes (a scale-invariant measure of prediction error), *r* is the Pearson correlation between predicted and observed outcomes, and *σpred/σtrue* quantifies how well the predicted variability matches that of the real data.

This formulation penalizes models that minimize MAE by collapsing variability (a common issue in regression with small samples) or that produce predictions with poor correlation with the target values despite low error. The 3 terms respectively ensure accuracy, directional fidelity, and variance realism, yielding models that generalize better across heterogeneous behavioral patterns. This multiterm objective extends beyond conventional MAE minimization by discouraging variance collapse, a frequent pitfall when small samples lead models to reproduce the mean rather than data variability.

By minimizing this composite objective, Optuna identifies the parameter configuration that provides the best trade-off between fit and generalization without over-regularizing or inflating variance. Once the optimal parameters are found, they are used consistently in the subsequent PEPHA stages for feature importance analysis, temporal order testing, and change point detection.

For the proof-of-concept application of PEPHA presented in this paper, Table 3 details the Optuna parameters explored across 500 tries. For the gamma range of values, “scale” is an adaptive γ based on feature variance; “auto” is a fixed *γ*=1/number of features, where γ is the kernel coefficient controlling how tightly the regressor fits around each training point (lower=smoother, higher=more localized).

The resulting optimal configuration was then used for the models described in the following sections, ensuring that feature importance and temporal analyses operated under the best validated parameter set.

**Table 3.** Optuna parameters explored with data from the Providemus alz project.

Parameter	Type of space of values	Possible values
MIN_VALID_WAVES	Integer	2 to 3
WINDOW_DAYS	Categorical	2, 3, 4, 5, 6, 7
USE_SE	Categorical	True, False
CORR_THRESHOLD	Categorical	0.60 to 1.00 (step=0.05)
SVR_C	Log uniform	1 to 10’000
SVR_epsilon	Log uniform	1 × 10–5 to 0.1
SVR_gamma	Categorical	“scale,” “auto,” 0.001, 0.005, 0.01, 0.05, 0.1, 0.5, 1.0

### Population Model and Feature Importance (Answering RQ1)

After generating slope-based predictors and deciding on the optimal complexity parameters to use, the goal of PEPHA is to estimate how strongly each 24-hour movement behavioral predictor potentially relates to the cognitive and mental health outcomes across the population and for an individual. This is done by training a regressive SVM model that maps all predictor features (linear and quadratic slopes, optional SE, and control variables that are reported once per wave, such as age or education) onto each of the outcomes of interest.

To avoid duplicated information conveyed by the predictor’s slopes and SEs, highly correlated predictor pairs (mean and median, IQR and SD, minimum and maximum, kurtosis and skewness) above a certain |ρ|=*CORR_THRESHOLD* in both halves have the second of them (median, SD, maximum, skewness) removed from the predictor set. Spearman rank correlation was chosen for this redundancy filtering step because it captures monotonic but potentially nonlinear relationships between predictors. In contrast, Pearson correlation, which is used in the Optuna parameter optimization, quantifies linear agreement on the continuous scale of the outcomes. This distinction ensures that redundancy removal remains sensitive to nonlinear codependencies, while model evaluation accurately reflects quantitative predictive power.

Key parameters controlling this stage include *CORR_THRESHOLD*, *SVR_C*, *SVR_epsilon*, and *SVR_gamma*.

Training then follows a leave-one-subject-out (LOSO) cross-validation (CV) strategy with *z*-normalization applied using training-fold statistics only (ie, normalizing per fold to avoid data leakage). Model accuracy is assessed using MAE, expressed in both natural units and as a percentage of the outcome’s scale (scaled MAE [SMAE]). For processing speed, SMAE values were obtained by dividing MAE by 14 (the empirical range between the minimum and maximum scores after filtering data within mean±2.5 SD), whereas for depressive symptoms values were divided by 22 (the theoretical range of the HADS).

Given the use of SVM as the PEPHA model, feature relevance was quantified via permutation importance, assessing the increase in prediction error when a given feature was randomly permuted. At this stage, averaging across all folds (all participants) to obtain the population ranking of features most predictive of the outcome, PEPHA provided a first-level answer to RQ1—identifying which aspects of 24-hour movement behavior most inform the resulting outcomes of processing speed and depressive symptoms.

### Personalized Timing Sensitivity via Temporal Analysis (Answering RQ2)

RQ2 asked whether the timing of behavioral change within each 90-day wave period was potentially associated with the processing speed and depressive symptoms at its end and whether individualized “potential temporal association windows” can be identified within each observation period.

In the PEPHA framework, this is operationalized through a half-swap analysis, in which predictors from the first and second halves of each wave (Half1 and Half2) are swapped to estimate how temporal order is linked to outcome predictions at the individual level. Note that the internal orders of both halves internal are maintained (Half1 from day 1 to 45 is kept in the same order 1 to 45). Thus, this test quantifies each participant’s sensitivity to when behavioral change occurs rather than how much change occurs. This sensitivity is interpreted as a marker of temporal association or responsiveness within the observation window, rather than as evidence that a behavior should occur at an exact time point in real-life intervention settings.

For each individual and wave, the model predicted the outcome twice: (1) using the original half-order (Half1 followed by Half2) and (2) using a swapped version in which early and late trends are interchanged (Half2 followed by Half1).

The difference between these 2 predictions,

$\Delta =y_{\text{observed}}-y_{\text{swapped}}$(2)

represents the individual’s timing sensitivity—how much the moment of behavioral change affects the expected outcome.

Statistical strength and direction of this effect were then summarized across waves for the same individual, using metrics such as the mean Δ, SD, Wilcoxon signed-rank test, Cohen *d* with bootstrap CIs (95% CI), and the probability of superiority (proportion of waves with *Δ*>0). These metrics quantified individual sensitivity of the outcome to the order of 24-hour movement behavior trends and changes, partially answering RQ2.

To illustrate how PEPHA operates at the individual level, we present 2 real participants in this proof-of-concept application, each analyzed for both outcomes: processing speed and depressive symptoms. These examples act as concise “use cases” showing how the framework uncovers personalized temporal-order dependencies between behavioral dynamics and outcomes.

Participants were selected based on the literature on cognitive aging and affective association, which consistently identify sex and age as the main drivers of change (women tend to have overall worse brain health at younger ages, but men’s decline is steeper with age [39-41]). Accordingly, we identified:

- Participant FY (female, younger): female, aged<50 years
- Participant MO (male, older): male, aged>60 years

### Within-Wave Change Point Detection and Intervention Timing (Answering RQ2)

To estimate when, within a wave, the largest behavioral shifts occur, PEPHA finally performs change point detection on the original daily sequences across waves of the same individual. Using the ruptures/pruned exact linear time (PELT) algorithm, change points are detected for each daily variable. It was selected because it provides an exact solution for multiple change point detection while maintaining near-linear computational complexity, allowing efficient analysis of daily behavioral time series where several structural shifts may occur within a wave. Such points are obtained via 2 cost models: (1) ℓ₂ (mean-shift), selecting the boundary with the largest Cohen *d* between segments, and (2) RBF (distributional-shift), detecting broader shape changes.

Each detected change point is characterized by its position (in days before the outcome assessment), magnitude of change, and data completeness. Aggregating these across waves yields individualized maps of potential “potential temporal association windows”—periods when outcomes may be most sensitive to changes in behavioral patterns. They signal candidate potential temporal association windows that may help prioritize when favorable or unfavorable configurations of behavior are likely to matter most (eg, reductions in sedentary time ≈ 20 d before assessment of processing speed), completing RQ2’s answer by revealing when behavioral timing is potentially most relevant for a given outcome and a given individual.

To operationalize PEPHA’s individualized component and for a proof-of-concept implementation, we applied such change point analysis to the top features with the highest permutation importance for a given participant and outcome. This approach enabled the identification of behavioral inflection points within each 90-day pre-assessment window.

Predictors were then organized according to groups. Within each, the mean number of days before assessment and its 95% CI were computed to identify clusters of potential temporal sensitivity (windows in which behavioral changes are most predictive of outcome variability).

Finally, to translate the detected change points into actionable temporal guidance, we visually overlapped all waves of the same individual for the 3 predictors with the highest importance for a given outcome. By aligning the daily trajectories relative to the outcome assessment, we examined how the behavioral pattern differs before versus after each detected change point. This step allowed interpretation of how the temporal ordering of behavioral patterns unfolds across waves, thereby informing how a potential halves-swap of the wave should be structured to reproduce the behavioral patterns associated with improved predicted outcomes per individual.

### Outputs and Interpretability

PEPHA produces 2 outputs at its end. At the population level, LOSO cross-validated SMAE and feature-importance tables identify the behavioral domains most predictive of the outcome studied. At the individual level, reports combine half-swap metrics (Δ, Cohen *d*, p, probability of superiority) with change point computations and confidence intervals. An illustration of PEPHA’s step-by-step methods is provided in Figure 3.

Together, these layers translate complex longitudinal data into personalized, temporally specific insights applicable to aging populations. PEPHA may offer a reproducible, interpretable basis for data-driven, timing-aware intervention planning, guiding future research toward individualized strategies that promote healthy trajectories of aging.

**Figure 3.**
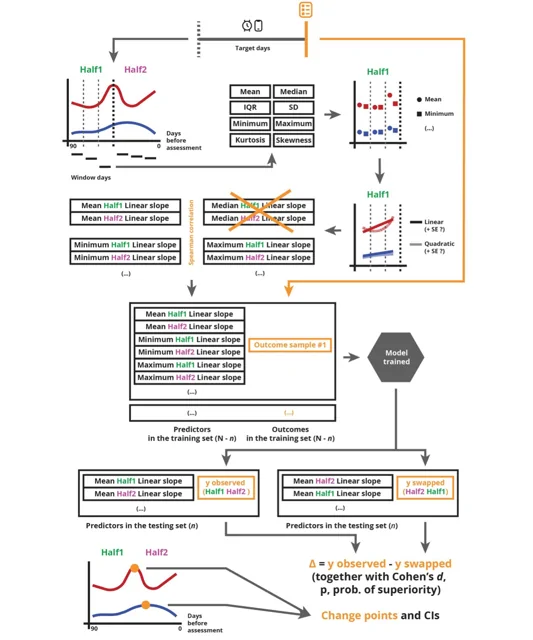
Diagram illustrating the PEPHA (Personalized Phenotyping for Aging) framework, from longitudinal data input to its outputs.

### Implementation Details

For the proof-of-concept application of the framework, all analyses were performed in Python 3.11 using standard scientific libraries, including pandas (2.2.2), NumPy (1.26), SciPy (1.13), scikit-learn (1.5), ruptures (1.1) for change point detection, and matplotlib (3.9) and seaborn (0.13) for visualization. Computations were executed on an Apple M1 Pro processor with 16 GB RAM running macOS 26, although the full pipeline is hardware-agnostic and compatible with both CPU- and GPU-based environments.

The code implementing the full PEPHA framework (from data preprocessing to feature extraction, modeling, temporal order testing, and change point analysis) was written to ensure full reproducibility. All configuration parameters (eg, window size, penalty for change point detection) were defined in a central location of the script, allowing flexible adaptation to other datasets and research contexts.

## Results

### Population-Level Performance

Given the extensive Optuna hyperparameter search performed for each outcome domain (see the Methods section), in this section, we report only the results obtained with the parameter configuration yielding the lowest composite Optuna objective function (see equation 1). Table 4 summarizes the most optimal complexity parameter values for the 4 model types: processing speed *levels*, processing speed *drift*, depression *levels*, and depression *drift*.

**Table 4.** Final optimal set of parameters found using Optuna for data from the Providemus alz project.

Parameter	PS^a^ levels	PS drift	Dep^b^ levels	Dep drift
MIN_VALID_WAVES	2	3	2	3
WINDOW_DAYS	2	6	6	6
USE_SE	False	False	False	True
CORR_THRESHOLD	0.60	0.75	0.60	0.75
SVR_C	361.7723	9981.6125	17.3368	4530.3347
SVR_epsilon	1.9990 × 10^-5^	0.0002	1.5604 × 10^-5^	1.2287 × 10^-5^
SVR_gamma	0.001	0.005	0.001	“auto”
Final objective value	0.5425	0.3094	0.3530	0.3311

a PS: processing speed.

b Dep: depressive symptoms.

The optimized parameters revealed systematic differences between the *levels* and *drift* variants of PEPHA. *Levels* models converged toward a minimum of 2 valid waves per participant, whereas *drift* models required at least 3 valid waves, consistent with the greater temporal stability needed to estimate changes in outcome trajectories. The temporal window size was predominantly 6 days, except for processing speed levels, which favored shorter 2-day windows, likely reflecting the higher short-term sensitivity of absolute processing speed performance to extreme fluctuations in 24-hour movement behavioral predictors. The inclusion of SE features did not generally improve model performance, except in the case of depression *drift*, where additional uncertainty information appeared to aid generalization.

*Levels* models tended to perform best with lower correlation thresholds for redundant feature removal (≈0.60), allowing moderately correlated predictors to remain, while *drift* models benefited from stricter pruning (≈0.75‐0.80). Similarly, *levels* models favored smaller support vector regression (SVR) regularization constants (C), promoting smoother fits, whereas *drift* models required larger C values to capture stronger nonlinear relations. No clear trends emerged for ε or γ.

As a result of the optimization by Optuna, the final objective formula value, its components, and the final N of participant data included in each finally built model are presented in Table 5.

**Table 5.** Final results after 10-fold cross-validation using the selected optimal parameters with Optuna for data from the Providemus alz project.

Parameters	PS^a^ levels	PS drift	Dep^b^ levels	Dep drift
MAEz	0.9874	0.9583	0.7342	0.9901
*r*	0.0415	0.1667	0.2356	0.1396
σpred/σtrue	0.8027	0.9393	0.4626	0.9929
Final objective value	0.5425	0.3094	0.3530	0.3311
Participants included, n	66	55	67	54

a PS: processing speed.

b Dep: depressive symptoms.

*Drift* models consistently achieved lower objective scores (see equation 1) than *levels* models. However, and due to the lower value of minimum waves required for inclusion, *levels* models preserved slightly broader population coverage (more included participants). Across outcomes, MAEz values were close to 1, consistent with the expected difficulty of forecasting cognitive functioning and mental health subtle signals in ecologically collected data.

Given that the primary objective of this paper was to introduce the PEPHA framework rather than to optimize predictive performance per se, we do not emphasize the relatively high MAEz values, serving as a proof-of-concept step only. In the next section, we report the final model performance for each outcome using the best-found hyperparameters and a LOSO CV protocol.

### Model Performance

As presented in Table 6, across all models, prediction errors remained within 8% and 20% of the range of values of the assessment tool (SMAE). The depression-related models achieved the lowest error rates, indicating that affective outcomes may exhibit more consistent relationships with wearable-derived 24-hour movement behavioral dynamics than cognitive performance does.

**Table 6.** Final cross-validated model performance using the Optuna-selected parameters and a leave-one-subject-out cross-validation.

	PS^a^ levels	PS drift	Dep^b^ levels	Dep drift
MAE^c^ (SD)	2.93 (2.27)	2.40 (2.07	1.77 (1.56)	2.97 (2.49)
SMAE^d^ (%), (SD)	20.90 (16.2)	17.11 (14.8)	8.05 (7.11)	13.52 (11.31)
*r*	0.0161	0.1642	0.2055	0.1004
σpred/σtrue	0.7716	0.9775	0.4735	1.0113

a PS: processing speed.

b Dep: depressive symptoms.

c MAE: mean absolute error.

d SMAE: scaled mean absolute error.

Performance patterns differed across outcomes and between the *levels* and *drift* variants. For processing speed, the *drift* model outperformed the *levels* model, yielding lower errors and higher correlation with observed values. Its σpred/σtrue ratio (0.98) indicated realistic variance reproduction, suggesting that gradual changes in behavioral habits across consecutive 90-day periods more effectively captured the dynamics underlying cognitive change than short-term fluctuations did.

For depressive symptoms, the opposite pattern emerged: The *levels* model achieved the lowest errors and the strongest correlation, indicating that short-term behavioral dynamics are more predictive of concurrent depressive levels. However, this model underestimated interindividual variability (σpred/σtrue=0.47), whereas the *drift* variant reproduced outcome dispersion almost perfectly (≈1.0) despite slightly higher errors.

In line with the study’s aims and RQs, the following sections focus exclusively on the *levels* models, as they directly address how changes in 24-hour movement behavior during the 90 days preceding an assessment relate to the observed outcome levels. The *drift* models, which capture habit changes across waves rather than immediate behavioral dynamics, will be explored in future work.

### Top 10 Features

Across both the processing speed and depressive symptoms outcomes, the 10 features whose permutation led to the largest SMAE increase are reported in Tables 7 and 8 and represent the most potentially influential predictors within their respective models.

**Table 7.** Top 10 features ranked by permutation importance for the *levels* model of processing speed.

Rank	Predictor	Group	Feature	Half	Trend	SMAE^a^ increase (%), mean (SD)
1	Sleep regularity index	Controls	Kurtosis	2	Linear	0.212 (0.775)
2	Minutes in MVPA^b^	Intensity-specific PA^c^ duration	Kurtosis	1	Linear	0.177 (0.850)
3	Seconds in intense activity	Intensity-specific PA duration	Kurtosis	1	Quadratic	0.155 (0.586)
4	Step frequency	Locomotor activity	Skewness	2	Linear	0.108 (0.812)
5	HR^d^ skewness	24-hour HR dynamics	IQR	1	Linear	0.108 (0.586)
6	Seconds in HR zone 3	Intensity-specific PA duration	Kurtosis	2	Quadratic	0.097 (0.872)
7	HR mean	24-hour HR dynamics	IQR	2	Linear	0.096 (0.389)
8	% missing windows	Controls	N/A^e^	N/A	N/A	0.086 (0.601)
9	Seconds in HR zone 2	Intensity-specific PA duration	Kurtosis	1	Quadratic	0.078 (0.331)
10	Seconds in HR zone 1	Intensity-specific PA duration	IQR	1	Linear	0.077 (0.583)

a SMAE: scaled mean absolute error.

b MVPA: moderate-to-vigorous physical activity.

c PA: physical activity.

d HR: heart rate.

e N/A: not applicable.

**Table 8.** Top 10 features ranked by permutation importance for the *levels* model of depressive symptoms.

Rank	Predictor	Group	Feature	Half	Trend	SMAE^a^ increase (%), mean SD
1	HR^b^ minimum	24-hour HR dynamics	Skewness	2	Quadratic	0.122 (0.454)
2	HR minimum	24-hour HR dynamics	Skewness	1	Quadratic	0.067 (0.374)
3	HR skewness	24-hour HR dynamics	Skewness	2	Quadratic	0.060 (0.294)
4	HR skewness	24-hour HR dynamics	Skewness	1	Linear	0.059 (0.237)
5	Seconds in HR zone 2	Intensity-specific PA^c^ duration	Minimum	1	Linear	0.057 (0.192)
6	HR skewness	24-hour HR dynamics	Kurtosis	2	Linear	0.053 (0.229)
7	HR IQR	24-hour HR dynamics	Mean	1	Quadratic	0.047 (0.204)
8	Step frequency	Locomotor activity	Skewness	2	Linear	0.045 (0.156)
9	Seconds in moderate activity	Intensity-specific PA duration	Minimum	2	Linear	0.044 (0.122)
10	Wearing time	Controls	Kurtosis	1	Quadratic	0.040 (0.218)

a SMAE: scaled mean absolute error.

b HR: heart rate.

c PA: physical activity.

When analyzing only the top 10 features for processing speed, most of them reflected the predictors in the intensity-specific PA duration group (5/10, 50%), were represented by kurtosis as the feature (5/10, 50%), and had linear trends as the predominant trend (60%). Interestingly, sleep regularity index was the top predictor.

Regarding depressive symptoms’ top 10 features, 24-hour heart rate dynamics was the group for the majority (6/10, 60%), with skewness the most represented feature (5/10, 50%) and linear and quadratic trends equally predominant. Control-related variables such as wearing time kurtosis also contributed modestly.

However, although the results presented in this section highlight only the 10 strongest predictors for each outcome, PEPHA’s design enables a broader examination of which behavioral and control dimensions systematically contributed to model accuracy across all features. The following section therefore expands this analysis to the full set of predictors, quantifying their overall importance and group-level representation within the population models.

### Distribution of Predictive Features

For processing speed, 144 features (29.63% of 486 total, based on the initial 8 features per 22 predictors sampled daily on each of the 2 halves and using linear and quadratic fits, added to the 7 predictors sampled once, and after removing the correlation-derived duplicates) yielded positive permutation values, indicating that their exclusion increased model error. Control variables such as education, total CRIq score, MDS, sex, subjective age difference, and chronological age did not appear among these positive contributors. Conversely, sleep regularity index, the proportion of missing windows, the number of tries on the Trail Making Test, and the wearing time were beneficial for modeling. For the other groups of predictors, all their variables contributed positively to prediction.

When grouped by the 24-hour movement behavioral domain, the proportional representation of informative predictors indicates locomotor activity, 24-hour heart rate dynamics, and intensity-specific PA duration as the most impactful groups for the modeling of processing speed across the population studied, as shown in Table 9.

Predictor-wise and considering the entire set of cases for which permutation led to a model performance drop (positive mean MAE increase), sleep regularity index (with a sum SMAE impact of 0.446% and 7% of the informative list features), heart rate skewness (0.327% and 9.2%, respectively), seconds spent in heart rate zone 3 (0.301% and 6.3%, respectively), and step frequency (0.297% and 7%, respectively) emerged as the most informative predictors. Analysis of the temporal features revealed a nearly balanced representation across halves (Half1: 50%; Half2: 48.6%; note that controls are not considered in this metric), with the second half achieving a higher sum of SMAE drop when permuted (1.886%) compared with the first half (1.763%). Quadratic trends contributed slightly more to the modeling (58.5% vs 40.1% for linear), with quadratic also obtaining a bigger model contribution (1.843% SMAE) compared with linear (1.763% SMAE). All parameters originated from slope-based representations, as SEs were not included in the best-performing processing speed configuration.

**Table 9.** Proportional representation and sum of scaled mean absolute errors (SMAEs) per group of predictors in the feature importance of the model of processing speed.

Predictor group	Proportional representation^a^, %	Sum of SMAEs^a^, %
Locomotor activity (n=2)	5.986	0.223
24-hour HR^b^ dynamics (n=8)	5.106	0.163
Intensity-specific PA^c^ duration (n=7)	4.326	0.180
Sedentary behavior (n=2)	1.408	0.007
Controls (n=3)	1.408	0.074

a Divided by the number of features in the group.

b HR: heart rate.

c PA: physical activity.

For depressive symptoms, 207 features (39.35% of 526) had positive permutation values. Like for processing speed, control variables such education, total CRIq, MDS, sex, and subjective age difference did not have predictive value. However, chronological age was important for the modeling of depressive symptoms. Additionally, the number of HADS tries did not appear among positive contributors, in contrast to Trail Making Tries tries for processing speed.

Table 10 contains the proportional representation and sum of SMAEs for each predictor group when modeling depressive symptoms.

**Table 10.** Proportional representation and sum of scaled mean absolute errors (SMAEs) per group of predictors in the feature importance of the model of depressive symptoms.

Predictor group	Proportional representation^a^, %	Sum of SMAEs^a^, %
Locomotor activity (n=2)	3.382	0.078
24-hour HR^b^ dynamics (n=8)	4.650	0.130
Intensity-specific PA^c^ duration (n=7)	4.969	0.092
Sedentary behavior (n=2)	5.797	0.118
Controls (n=3)	0.918	0.025

a Divided by the number of features in the group.

b HR: heart rate.

c PA: physical activity.

Across the list of informative predictors, the predictors with the highest impact on the model’s performance were heart rate minimum (0.332% of the difference in SMAE and 6.3% of the informative features list), heart rate skewness (0.227% and 3.9%, respectively), and seconds spent in heart rate zone 2 (0.205% and 6.3%, respectively). A similar proportion of informative features originated from each half of the wave (Half1: 51.2%; Half2: 47.8%, with sums of SMAEs being 1.107% and 1.219%, respectively), suggesting that late-period dynamics of activity may carry stronger affective relevance. Like processing speed, the depression model did not benefit from the inclusion of SE parameters. Linear trends were mostly as represented (48.8%, sum MAE difference 1.087%) as the quadratic ones (50.2% and 1.239%, respectively).

### Personalized Predictions and Order-Swap Analysis

This section focused on answering RQ2—whether the timing of behavioral change within each 90-day wave period could be linked to the processing speed and depressive symptoms at its end. It does so by searching for the potential temporal association windows per participant and outcome case, thus providing an individualized result and insight.

#### Aggregate Order-Swap Effects

Across all participants, temporal order effects were detected for both outcomes, as summarized in Table 11. For processing speed, the near-zero (but still positive) mean *d* coupled with high variability indicates that reversing the order of behavioral halves had little overall population effect but large interindividual differences (some participants benefited from behavioral shifts, and some did not). For depressive symptoms, the mean negative *d* and similarly high dispersion indicate equally heterogeneous but partially opposing patterns, with the negative mean indicating an average increase of the outcome value when swapping halves of the wave.

**Table 11.** Group-level summary of temporal order effects estimated through the order-swap anlysis in PEPHA (Personalized Phenotyping for Aging).

Population result	Processing speed	Depressive symptoms
Cohen *d*, mean (SD)	0.051 (0.784)	–0.120 (0.942)
|*d*|>0.5, n (%)	27 (40.91%)	29 (43.94%)
Probability of superiority, mean (SD)	0.508 (0.269)	0.468 (0.212)
Wilcoxon *P* value, mean (SD)	.50 (.31)	.52 (.29)
Halves outcome Δ, mean (SD)^a^	0.208 (1.787)	–0.062 (0.798)
Halves outcome Δ (%), mean (SD)^a^	1.484 (12.762)	–0.440 (5.698)

a Informed the Δ values in the scaled mean absolute error (SMAE).

Roughly 40% to 45% of participants exhibited medium or large effects (|*d*|>0.5), confirming that potential temporal sensitivity was substantial for a considerable subset of individuals even when group-level averages were near zero. Although Wilcoxon *P* values were not consistently significant due to the limited number of waves per participant (maximum of 5), the distribution of effect sizes supports the presence of meaningful within-person temporal dependencies.

#### Illustrative Individual Pattern

The 2 illustrative participants (FY and MO) were chosen among individuals whose models achieved SMAE <10% under LOSO CV, ensuring that their predictions were robust enough to allow meaningful interpretation of order-swap effects. This 10% threshold was adopted because, although depressive symptom models occasionally achieved errors ≤5%, no processing speed case met that stricter criterion; using the same 10% cutoff for both outcomes thus provided a balanced and comparable basis for illustration. Table 12 summarizes demographic and model-derived characteristics for both participants across outcomes.

**Table 12.** Characteristics and individualized PEPHA (Personalized Phenotyping for Aging) results from the leave-one-subject-out cross-validated model predictions for the 2 illustrative participants: female, younger (FY) and male, older (MO).

Individual result	FY PS^a^	FY Dep^b^	MO PS	MO Dep
Age at enrollment (years)	46.17	46.17	63.78	63.78
Sex	Female	Female	Male	Male
Total CRIq^c^ score	143.00	143.00	135.00	135.00
Scaled MAE^d^	7.94%	9.36%	8.78%	6.25%
Cohen *d* (95% CI)	0.281 (–0.712 to 1.448)	0.627 (–0.246 to 2.909)	0.706 (0.315 to 3.431)	–0.261 (–5.451 to 0.907)
Probability of superiority	0.60	0.60	0.8	0.25
Δ, mean (SD)	0.568 (2.020)	1.177 (1.876)	1.174 (1.663)	–0.665 (2.545)
Scaled Δ (%), mean (SD)	4.05 (14.43)	5.35 (8.53)	8.39 (11.88)	–3.02 (11.57)
Waves in test set, n	5	5	5	4
Wilcoxon *P* value	.47	.44	.13	.63

a PS: processing speed.

b Dep: depressive symptoms.

c CRIq: Cognitive Reserve Index questionnaire.

d MAE: mean absolute error.

SMAE values for both participants (FY and MO) ranged between 6% and 9% of the respective assessment tool ranges, confirming comparable and acceptable prediction accuracy under LOSO CV.

When examining the mean Cohen *d* values, only 2 models exceeded the conventional medium-effect threshold of |*d*|>0.5: depression in the FY participant (*d*=0.627) and processing speed in the MO participant (*d*=0.706). However, CIs revealed that, despite the small number of test waves (4 to 5 per participant), only the MO processing speed case maintained a fully positive 95% CI (0.315 to 3.431). This consistency suggested a high probability that swapping the temporal order of behavioral halves would potentially impact the predicted processing speed score but only at these specific individual cases.

Such interpretation aligns with the probability of superiority, where only MO processing speed showed a value substantially above chance (0.80), while all other conditions hovered around chance (0.60) or below (0.25). The Wilcoxon *P* value of .13, although not statistically significant due to the limited number of test waves, was the lowest among all conditions and directionally consistent with the larger effect size and superiority metrics.

Taken together, these results indicate that only the more vulnerable participant (MO) displayed a clear temporal order sensitivity and that this sensitivity was evident only for processing speed. In contrast, depressive symptoms for both participants—and processing speed in the less vulnerable case—showed no systematic differences when behavioral halves were swapped, indicating a potential order insensitivity and temporal stability, at least with the currently existing data.

As a proof of concept, these findings provide a partial answer to RQ2, demonstrating that PEPHA can identify individualized timing effects even within small-wave longitudinal data. However, such conclusions are not possible and not intended to be generalized at the population level, as that is not the intended use of PEPHA. To further illustrate its interpretive potential, the following section focuses on the MO participant’s processing speed model, exploring the next methodological step of PEPHA (change point detection) to estimate when, within the 90-day window, behavioral variations are most likely to influence processing speed for that individual.

### Change Point and Temporal Importance Results

In the individualized analysis of processing speed for participant MO, the behavioral dimensions highlighted differed from the population-level pattern. Among the top 10 features (Table 13), 24-hour heart rate dynamics accounted for one-half of the features, instead of intensity-specific PA duration, which was the most relevant group at the population level. Although population results did not reveal a predominant half in the top 10 features, for the participant MO’s case Half 2 was represented in 70 % of the top features, displaying a higher relevance of the second 45-day period in the outcome.

**Table 13.** Top 10 features ranked by permutation importance for the *levels* model of processing speed for the cross-validation fold in which male, younger (MO) was the test participant.

Rank	Predictor	Group	Feature	Half	Trend	SMAE^a^ increase (%), mean (SD)
1	HR^b^ minimum	24-hour HR dynamics	IQR	2	Quadratic	1.129 (0.464)
2	Seconds in intense activity	Intensity-specific PA^c^ duration	Minimum	2	Quadratic	1.114 (0.764)
3	HR median	24-hour HR dynamics	Kurtosis	2	Quadratic	1.093 (0.593)
4	HR SD	24-hour HR dynamics	Skewness	1	Quadratic	0.929 (0.579)
5	Distance frequency	Locomotor activity	Kurtosis	2	Linear	0.800 (0.507)
6	Seconds in HR zone 1	Intensity-specific PA duration	Maximum	1	Quadratic	0.736 (0.307)
7	Seconds in HR zone 1	Intensity-specific PA duration	IQR	2	Quadratic	0.714 (0.829)
8	Seconds in soft activity	Intensity-specific PA duration	IQR	1	Quadratic	0.650 (0.386)
9	HR minimum	24-hour HR dynamics	Minimum	2	Quadratic	0.621 (0.393)
10	HR kurtosis	24-hour HR dynamics	Kurtosis	2	Linear	0.529 (0.771)

a SMAE: scaled mean absolute error.

b HR: heart rate.

c PA: physical activity.

Recalling the positive Cohen *d* observed earlier for this individual, swapping the 2 halves of the behavioral window would lead to a decrease in the processing speed score of this individual, corresponding to improved performance (since lower values reflect faster completion times).

To examine when such adjustments might be most effective, we overlapped the 95% CIs of all the predictors’ change points detected according to our methods. The per-predictor plots presented in Figures 4 and 5 show the distribution of individual change points, with the bars indicating the 95% CI for each predictor for which a change point was detected and red markers representing the mean. Cohen *d*–based change points identify the time within the observation window at which the magnitude of behavioral change between earlier and later segments is largest, independent of outcome relevance. In contrast, RBF-derived change points reflect moments when behavioral shifts most strongly influence the model’s predicted outcome. Thus, the former captures maximal behavioral differences, whereas the latter captures maximal outcome-relevant differences.

The aggregated distribution (Figures 6 and 7) revealed a mean change point at 40.5 days before the assessment (median 38.0, 95% CI 26.4 to 62.1) based on Cohen *d* and 45.9 days before the assessment (median 48.0, 95% CI 30. to 54.5) based on the RBF-derived importance scores.

**Figure 4.**
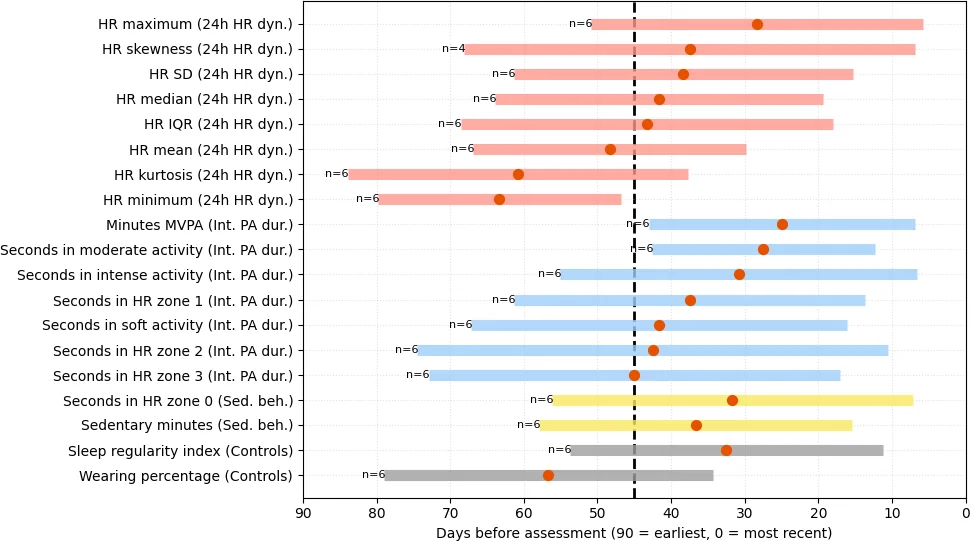
Illustrative example of change point distributions per predictor based on Cohen *d* (original halves’ order) for the participant male, younger (MO), grouped according to predictor group and sorted according to mean change point. Locomotor activities did not achieve the minimum of 2 change points detected to derive a CI. dyn.: dynamics; HR: heart rate; Int. PA dur.: intensity-specific physical activity duration; MVPA: moderate-to-vigorous physical activity; Sed. beh.: sedentary behavior.

**Figure 5.**
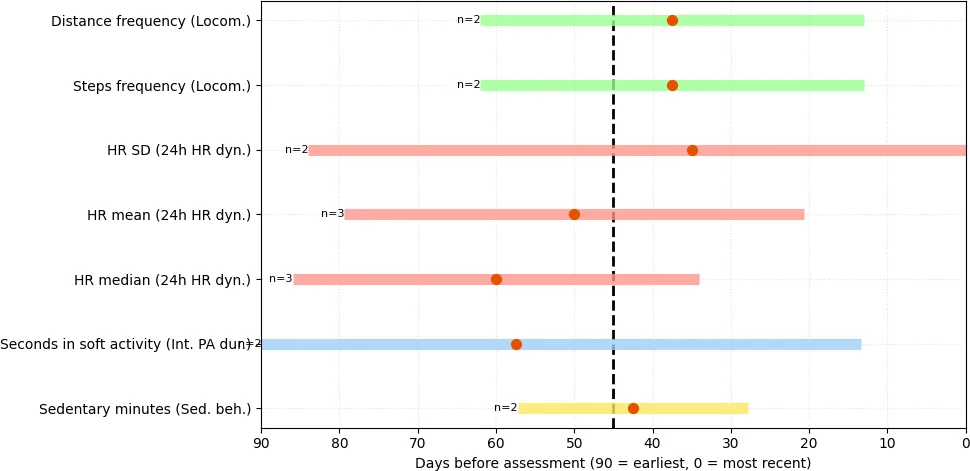
Illustrative example of change point distributions per predictor based on radial basis function (RBF) cost (original halves’ order) for the participant male, older (MO), grouped according to the predictor group and sorted according to mean change point. Controls did not achieve the minimum of 2 change points detected to derive a CI. dyn.: dynamics; HR: heart rate; Int. PA dur.: intensity-specific physical activity duration; Locom.: locomotor activity; Sed. beh.: sedentary behavior.

**Figure 6.**
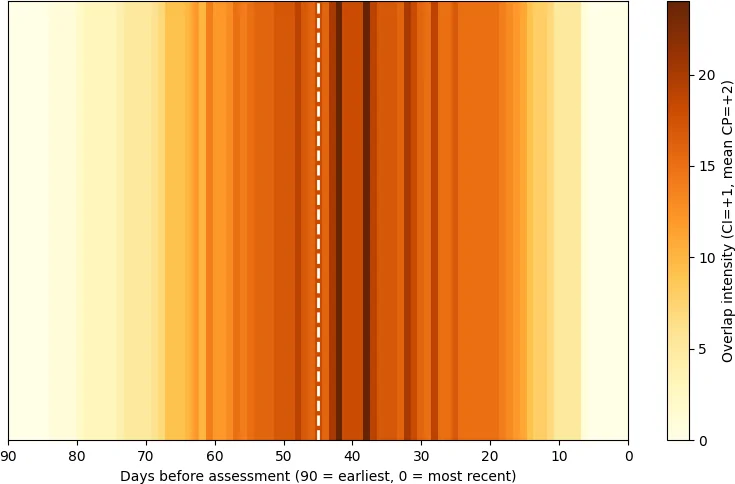
Illustrative example of aggregate overlap of change point CIs in the originally observed data using Cohen *d* for the participant male, older (MO).

**Figure 7.**
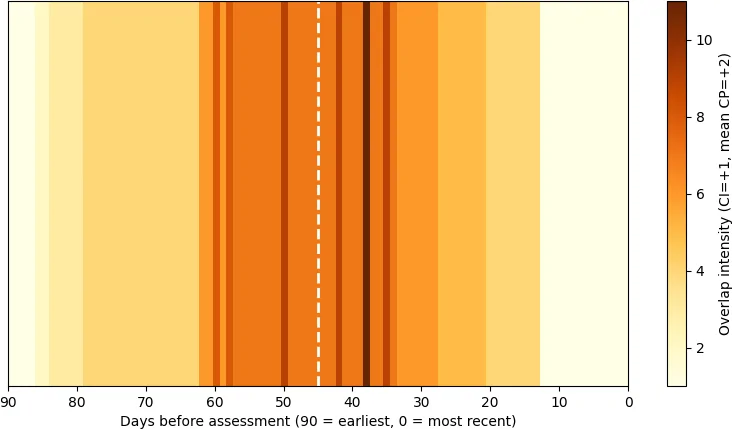
Illustrative example of the aggregate overlap of change point CIs in the originally observed data using radial basis function (RBF) for the participant male, older (MO).

When analyzing aggregate overlaps of detected change points for only the most informative group of predictors (24-hour heart rate dynamics), the results (Figure 8) obtained with Cohen *d* revealed a mean change point at 45.1 days before assessment (median 42.5, 95% CI 29.8 to 62.6) and with RBF at 48.3 days before assessment (median 50.0, 95% CI 35.8 to 59.5).

However, after knowing that MO processing speed would benefit from a halves-swap of their behavior data and knowing the change point across waves of the top 3 predictors of their processing speed, one final question remains: how should that change happen? For example, should the IQR of the minimum 24-hour heart rate increase or decrease after the change point of 63.3 days before the outcome assessment?

To answer that final step, Figure 9 presents the overlap of the 5 waves of MO’s data for the top 3 features as well as their evolution over time.

**Figure 8.**
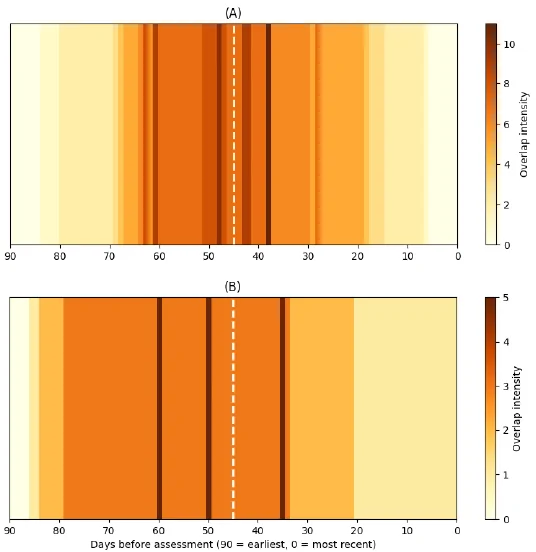
Illustrative example of the aggregate overlap of change point CIs in the originally observed 24-hour heart rate dynamics data using (A) Cohen *d* and (B) radial basis function (RBF) for the participant male, older (MO).

**Figure 9.**
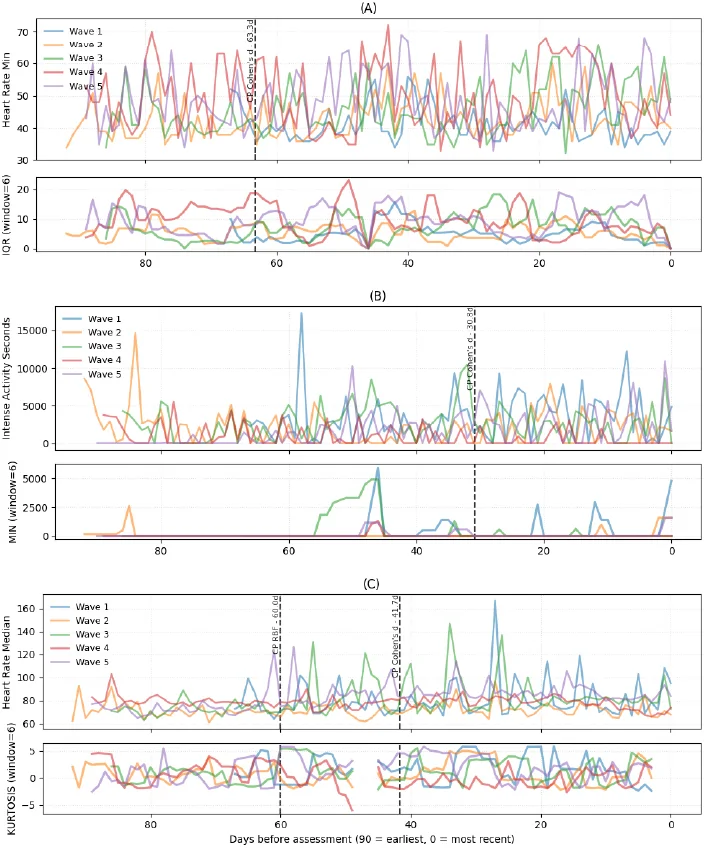
Illustrative example of the wave’s data and computed metrics in the top 3 importance features of the processing speed for participant male, older (MO), with vertical lines representing the change points identified by Personalized Phenotyping for Aging (PEPHA): (A) heart rate minimum, (B) intensity activity (seconds), (C) heart rate median.

For the strongest predictor, the IQR of daily minimum heart rate, the wave-overlap plots (Figure 9A) indicate that, before this change point (earlier in the wave, further from the assessment), the values were generally lower and more stable across waves. After the change point (closer to the assessment), the IQR increased and became more variable. This pattern suggests a transition from relatively stable nocturnal/resting cardiovascular dynamics earlier in the wave toward greater dispersion closer to the assessment. Importantly, the prior order-swap result indicated that reversing the temporal order of the 2 halves improved MO’s predicted processing speed. This suggests that performance would benefit if the more stable, low-IQR pattern occurred closer to the assessment (26 d to 0 d prior) while the more variable pattern occurred earlier in the wave (90 d to 26 d prior).

For the second strongest predictor, the minimum of daily seconds spent in intense activity (Figure 9B), the results indicated that, earlier in the wave, the minimum values remained low overall but with fewer and more sustained increases visible as extended plateaus rather than brief excursions. Later, they were generally low but punctuated by frequent, short spikes, indicating intermittent engagement in higher-intensity activity. This pattern reflects a shift from a more sustained (but temporally constrained) intense activity presence earlier to an irregular, burst-like exposure later in the wave toward. The order-swap results suggested that the relevant adjustment was, again, not a simple increase or decrease in intense activity but rather a reorganization of its timing. Specifically, positioning the earlier-wave pattern of more sustained minimum intensity closer to the assessment (60 d to 0 d before) and shifting the later-wave pattern of brief, irregular spikes to an earlier period (90 d to 60 d) would be associated with improved processing speed performance. For the third strongest predictor, the kurtosis of the daily median heart rate (Figure 9C), 2 change points were detected: one at 41.7 days before the assessment using Cohen *d* and another at 60.0 days before the assessment using the RBF-derived importance scores.

The wave-overlap plots show that, relative to the Cohen *d* change point, earlier in the wave, the kurtosis values were generally lower and less variable, indicating heart rate distributions closer to a more regular, light-tailed shape. Later in the wave (closer to the assessment), kurtosis increased and exhibited greater variability, reflecting more peaked and heavy-tailed distributions with occasional extreme values. In contrast, the RBF-derived change point at 60 days primarily separated periods by the maximum attained kurtosis, with higher extreme values occurring predominantly closer to the assessment. Together, these results indicate a transition from more regular, stable heart rate distribution shapes earlier in the wave toward increasingly irregular and extreme dynamics later. The order-swap results suggest that shifting the lower kurtosis, less variable regime to the later portion of the wave (49 d to 0 d before the assessment) and relocating the higher, more variable kurtosis patterns to the earlier portion (30 d to 60 d before) would be associated with improved processing-speed performance.

Together, these complementary analyses indicate that MO’s processing speed performance was most sensitive not to absolute levels of PA or heart rate metrics but to their temporal ordering within each wave. Across the 3 strongest predictors (variability in minimum heart rate, minimum engagement in intense activity, and the distributional shape of median heart rate), a consistent pattern emerged: Configurations characterized by greater stability, regularity, or sustained structure were beneficial when they occurred closer to the assessment, whereas more variable, burst-like, or extreme patterns were better positioned earlier in the wave. The change point analyses identified when these configurations naturally shifted, while the order-swap result clarified the direction of benefit, indicating that reversing the observed before/after sequence would potentially improve processing speed. Taken together, these findings suggest that, for MO, the pattern associated with improved predicted processing speed was characterized by a temporal reorganization of existing 24-hour movement behavioral patterns, in which stable cardiovascular dynamics and more sustained activity engagement were positioned closer to the assessment, rather than by uniform increases or decreases in activity or heart rate levels.

## Discussion

### Principal Findings

This paper introduced and empirically validated PEPHA, an interpretable AI framework that provides a way to identify individual potential temporal association windows and the behavioral dimensions that contribute to health-relevant outcomes. PEPHA is purposefully hypothesis-generating rather than causal: It surfaces who, which behavioral dimensions, and during which periods outcomes appear more sensitive to behavioral variation. Importantly, these windows should not be interpreted as indicating that a person should begin a behavior at an exact number of days before an assessment but rather as identifying periods of increased association or responsiveness within which favorable or unfavorable behavioral patterns may matter more.

At the population level and using data from the Providemus alz study, PEPHA highlighted distinct temporal and behavioral conclusions for processing speed and depressive symptoms. Processing speed was characterized by a broad contribution of locomotor activity, 24-hour heart rate dynamics, and intensity-specific PA duration, with nonlinear temporal patterns playing a prominent role. This suggests that cognitive functioning in aging is potentially associated not only with overall levels of 24-hour movement behavior but also with how behavioral and physiological signals fluctuate and evolve within an observation period prior to assessment. In contrast, depressive symptoms were primarily associated with sedentary behavior alongside intensity-specific PA duration and heart rate dynamics, with greater relevance of 24-hour movement behavioral patterns occurring later within the observation window (second half of the 90 d prior to the assessment). Together, these findings underscore that cognitive functioning and mental health in aging are potentially linked to similar configurations of behavioral dynamics but in different relevance order, emphasizing the importance of temporal distribution and patterning of 24-hour movement behaviors rather than aggregate activity alone. However, the empirical findings presented here primarily serve to illustrate the framework’s analytical capabilities rather than to establish novel relationships between PA and cognition or mental health or to provide clear intervention plans to be implemented.

Beyond identifying which behavioral dimensions matter on a population level, PEPHA also served to uncover when behavioral change appears most consequential individually. Although not finding a clear trend in the population-aggregated analysis, the framework isolated participant-specific “potential temporal association windows” (ie, periods where altering the sequence or stability of 24-hour movement behaviors most influenced predicted outcomes). To demonstrate this individualized component, 2 real participants (female<55 years old; male, >60 years old) were analyzed as illustrative cases. Despite comparable prediction accuracy, only the male participant exhibited a consistent, meaningful effect of temporal order, indicating that behavioral sequencing played a role in the cognitive outcome (estimated 8% decrease in predicted processing speed scores when the halves are swapped, suggesting that a change in behavior order was potentially associated with an increase of cognitive performance). For this individual, PEPHA localized the main window of sensitivity to change approximately 60 days before the cognitive assessment for 2 physiological signals (heart rate minimum and median) and 30 days for intense activity duration, suggesting that this individual’s processing speed may have been particularly sensitive to favorable or unfavorable behavioral configurations during those periods. Rather than indicating that a specific behavior should be initiated exactly 30 days or 60 days before assessment, these findings point to windows of increased potential temporal association in which the sequencing and stability of 24-hour movement behavior may be more relevant to the outcome. Future population-wide analyses will extend this approach across individuals, enabling systematic characterization of how such individualized “potential temporal association windows” vary as a function of demographic, cognitive reserve, and clinical profiles.

From a practical perspective, PEPHA is intended to support personalized interpretation rather than automated clinical decision-making. For example, a clinician could use the framework to identify which behavioral dimensions appear most relevant for an individual and whether these associations are concentrated within specific periods preceding an assessment. These outputs could support personalized monitoring, support personalized behavior change trials, guide conversations regarding lifestyle patterns, and generate individualized hypotheses for future evaluation. Importantly, they should not be interpreted as behavioral prescriptions or recommendations regarding intervention timing but rather as interpretable summaries of personalized temporal associations requiring prospective validation.

Although the absolute prediction errors can still be considered large (an expected property of ecological data collected in free-living settings), the proposed framework achieved its primary goal on this first proof-of-concept application: to derive individualized, interpretable analyses of 24-hour movement behaviors capable of generating personalized hypotheses regarding the temporal relevance of behavioral patterns for healthy aging. This implementation was not compared against nontemporal behavioral representations, as its objective was not to demonstrate superiority over existing predictive models but to establish the feasibility and interpretability of a temporal modeling framework. Future work should directly quantify the incremental value of incorporating temporal information through comparisons with nontemporal baselines and more complex temporal architectures.

### Limitations

Nevertheless, this first empirical application of PEPHA should be interpreted considering several methodological and data-related limitations. First, the proof of concept relied on the Providemus alz dataset, which, despite its unique longitudinal high-frequency nature, comprised a relatively small and demographically homogeneous sample. Consequently, population-level estimates may underrepresent interindividual diversity in PA patterns; cognitive baselines; and contextual factors such as work status, comorbidities, or seasonal variability. Extending PEPHA to larger and more heterogeneous cohorts will be essential to establish the generalizability of its individualized findings.

Second, the number of repeated outcome assessments per participant was limited (3 to 5), which constrains the precision of temporal order and change point estimates. The observed wide CIs for individual-level metrics such as Cohen *d* or the probability of superiority reflect this inherent uncertainty. Consequently, the individualized behavioral patterns and temporal association windows presented in this work should be interpreted as illustrative applications of the PEPHA framework rather than definitive evidence of stable person-specific temporal effects. Their robustness and generalizability should be evaluated in future studies with substantially longer longitudinal follow-up and a greater number of repeated assessments per participant. Such datasets will enable more reliable estimation of individualized temporal patterns as well as formal evaluation of their reproducibility under different modeling assumptions.

Third, this first implementation of PEPHA deliberately prioritized interpretability over maximal temporal representational power. By summarizing behavioral trajectories using linear and quadratic temporal trends, the framework produced compact and clinically interpretable descriptors of behavioral evolution that can be directly related to individual outcomes. To minimize the inevitable trade-off between interpretability and information preservation, the framework summarized each temporal window using a set of descriptive statistical metrics (eg, central tendency, variability, distributional shape) that have been widely adopted in previous wearable-sensing and digital phenotyping research to characterize behavioral and physiological time series. Nevertheless, this design inevitably sacrifices part of the temporal richness present in continuous wearable data. In particular, higher-order temporal characteristics such as circadian organization, burst-like behavioral episodes, recurrent patterns, and complex nonlinear transitions may not be fully preserved by the current feature representation. We intentionally accepted this trade-off in this first implementation because the primary objective was to demonstrate an interpretable methodological framework capable of generating individualized temporal hypotheses rather than maximizing predictive performance. Future work should investigate richer temporal representations, including sequence-based architectures such as long short-term models (LSTMs) or transformer models combined with explainability techniques (eg, temporal SHAP values or attention-based interpretation), to determine whether additional temporal information can be exploited while maintaining clinically meaningful interpretability. Such approaches, however, require substantially larger longitudinal datasets than those available for this initial proof-of-concept implementation and would, therefore, fall out of scope of this initial research.

Finally, this first proof of concept tested the framework on cognitive functioning and mental health outcomes derived from ecological self-assessments and mobile digital games. These measures, though validated, remain subject to within-person noise and daily fluctuations. Integrating additional objective or clinical endpoints (eg, biomarkers or mobility indicators) could strengthen external validity and clarify the mechanistic pathways linking behavior and brain health.

Overall, these limitations do not detract from the framework’s conceptual contribution. Instead, they outline a clear research agenda: scaling PEPHA to multisite datasets, integrating multimodal signals, extending it to other health outcomes, extending it to randomized control trial implementation for causality analysis of its results, and closing the loop between individualized behavioral prediction and adaptive, real-world intervention design.

In the context of healthy aging, the PEPHA approach supports a paradigm in which AI no longer prescribes uniform recommendations but instead derives personalized “potential temporal association windows.” These can inform hypotheses about intervention timing, periods of increased sensitivity, and the tailoring of PA guidance to each person’s physiological and lifestyle rhythms. Such personalization directly responds to the need of using AI for tailoring PA interventions based on multidimensional, real-world data.

The framework’s modular structure also ensures its transferability beyond the current use case. Its parameterization, such as window length, feature selection thresholds, and outcome types, can be adapted to diverse datasets, from clinical PA trials to community-based monitoring studies. The framework’s interpretability further supports clinical translation, allowing practitioners to visualize how behavioral dynamics influence health trajectories and to communicate AI-derived recommendations transparently to older adults.

In summary, PEPHA lays the groundwork for a new generation of explainable and actionable AI tools that bridge observational sensing and behavioral intervention. By enabling individualized insight into both the mechanisms and timing of behavior-health relationships, it aligns with the broader goal of transforming digital health data into personalized, adaptive strategies that promote functional capacity, autonomy, and quality of life in aging populations.

### Conclusions

In this article, we presented and applied a novel framework, with which we demonstrated that naturally occurring 24-hour movement behavior can reveal both which behavioral dimensions and when changes most potentially influence cognitive functioning and mental health in adults.

In addition to population-derived conclusions, PEPHA allowed us to study the individual level, detecting unique and personalized “potential temporal association windows” and showing that behavioral order and timing can modulate cognitive and mental health outcomes, particularly in specific aging profiles. The empirical findings should be interpreted as illustrative applications of the framework rather than definitive evidence of novel behavioral associations.

Although preliminary, these findings illustrate how interpretable AI can move beyond prediction to prescription, transforming high-frequency observational data into actionable, personalized hypotheses for optimizing PA in everyday life. Future work must expand PEPHA to larger, more heterogeneous samples and evaluate its integration into adaptive intervention platforms, ultimately contributing to the development of explainable, data-driven, and timing-sensitive PA personalization for healthy aging.

## Abbreviations

AI: artificial intelligence
CRIq: Cognitive Reserve Index questionnaire
CV: cross-validation
FY: female, younger
HADS: Hospital Anxiety and Depression Scale
LOSO: leave-one-subject-out
LSTM: long short-term model
MAE: mean absolute error
MDS: Mediterranean diet score
MO: male, older
MoTR: model-twin randomization
PA: physical activity
PELT: pruned exact linear time
PEPHA: Personalized Phenotyping for Aging
RBF: radial basis function
SMAE: scaled mean absolute error
SVM: support vector machine
SVR: support vector regression
